# Physical properties and biological effects of ceramic materials emitting infrared radiation for pain, muscular activity, and musculoskeletal conditions

**DOI:** 10.1111/phpp.12799

**Published:** 2022-05-21

**Authors:** Jan Kyselovic, Jozef Masarik, Hayet Kechemir, Eva Koscova, Iva Igracki Turudic, Michael Richard Hamblin

**Affiliations:** ^1^ Clinical Research Unit, 5th Department of Internal Medicine, Faculty of Medicine Comenius University, University Hospital Bratislava Bratislava Slovak Republic; ^2^ Department of Nuclear Physics and Biophysics, Faculty of Mathematics, Physics, and Informatics Comenius University Bratislava Bratislava Slovak Republic; ^3^ Consumer Healthcare Medical Affairs Department, Sanofi CHC Paris France; ^4^ Consumer Healthcare Medical Affairs Department Bratislava Slovakia; ^5^ Consumer Healthcare Medical Affairs Department, Sanofi CHC Frankfurt Germany; ^6^ Laser Research Centre, Faculty of Health Science University of Johannesburg Johannesburg South Africa

**Keywords:** bioceramic, infrared, mechanism of action, musculoskeletal pain

## Abstract

**Background:**

Up to 33% of the general population worldwide suffer musculoskeletal conditions, with low back pain being the single leading cause of disability globally. Multimodal therapeutic options are available to relieve the pain associated with muscular disorders, including physical, complementary, and pharmacological therapies. However, existing interventions are not disease modifying and have several limitations.

**Method:**

Literature review.

**Results:**

In this context, the use of nonthermal infrared light delivered via patches, fabrics, and garments containing infrared‐emitting bioceramic minerals have been investigated. Positive effects on muscular cells, muscular recovery, and reduced inflammation and pain have been reported both in preclinical and clinical studies. There are several hypotheses on how infrared may contribute to musculoskeletal pain relief, however, the full mechanism of action remains unclear. This article provides an overview of the physical characteristics of infrared radiation and its biological effects, focusing on those that could potentially explain the mechanism of action responsible for the relief of musculoskeletal pain.

**Conclusions:**

Based on the current evidence, the following pathways have been considered: upregulation of endothelial nitric oxide synthase, increase in nitric oxide bioavailability, anti‐inflammatory effects, and reduction in oxidative stress.

## 1 INTRODUCTION

Musculoskeletal pain refers to a number of conditions that affect the locomotor system of individuals, including bones, muscles, ligaments, tendons, and nerves.^1^ There are more than 200 musculoskeletal conditions ranging from disorders such as neck or back pain to autoimmune diseases like rheumatoid arthritis.^2^ The pain caused by these conditions can be either acute or chronic and is characterized by a diffuse or focal painful sensation in musculoskeletal or associated neural tissues.^3^ In addition to pain, common symptoms of musculoskeletal disorders are stiffness or ache, fatigue, and burning sensations in the muscles. Symptoms may progressively increase with exacerbation of tissue injury and inflammation in affected anatomical sites.^3^, ^4^ Musculoskeletal pain is most often the consequence of cumulative traumatic injury (abrupt jerking movements, falls, sprains), repetitive strain, or overuse of muscles. Pain can also develop as a consequence of neuropathy, tendonitis, tendinosis, myalgia, and even stress fractures.^1^, ^4^ The pathophysiology of musculoskeletal pain is not yet fully understood, but some mechanisms of inflammation, fibrosis, tissue degradation, and neurosensory disturbance have been described.^4^ Worldwide, about 1.71 billion people have musculoskeletal conditions.^1^ Musculoskeletal conditions are the leading contributory factor causing disability globally, with low back pain being the single leading cause of disability.^1^ These disorders constitute an enormous burden to society in terms of suffering, lost productivity, and healthcare costs (which have doubled since 2010).^1^, ^5^ Musculoskeletal pain is frequently managed in the primary care setting. Therapeutic options include physical modalities (e.g., exercise therapy), complementary therapies (e.g., acupuncture), and pharmacological interventions (e.g., analgesics, nonsteroidal anti‐inflammatory drugs [NSAIDs], and corticosteroid injections).^6^ In some cases, surgical interventions may also be considered. Noteworthy, the existing therapies are useful for treating the symptoms, but they are not disease modifying and do not address the underlying cause of pain.

Infrared radiation is a band of the electromagnetic spectrum, the wavelength of which ranges between visible light (0.4–0.7 μm) and microwaves (1000 μm–1 m).^7^ The use of infrared radiation has been investigated for different medical applications, ranging from diagnosis to therapy (including musculoskeletal pain). One of the oldest treatments using infrared radiation is the infrared sauna therapy, or “Waon therapy,” which has been used extensively in Japan and Korea to improve cardiac and vascular function and to reduce oxidative stress in patients with chronic heart failure.^8^, ^9^, ^10^ In the past decades, developments in physics and technology have increased the scope of infrared radiation for therapeutic use,^11^ such as infrared heat lamps which have been widely used both in at‐home settings for painful conditions and by physical therapists for sports injuries or orthopedic conditions.^12^ Many studies have reported the positive effects of infrared on cells and tissues,^13^ and the clinical benefits for patients,^11^ however, there have been few peer‐reviewed studies describing the mechanism of action or the results of controlled clinical trials for pain relief in patients with musculoskeletal conditions.

The aim of this article is to provide an overview of the physical characteristics of nonthermal infrared radiation, more specifically the infrared radiation that falls within the far waveband (3–1000 μm; IR‐C), and its biological effects, focusing on those that could potentially explain the mechanism of action responsible for the relief of musculoskeletal pain. An overview of the current infrared medical applications based on the use of patches, fabrics, and garments containing infrared‐emitting bioceramic minerals that emits IR‐C radiation at body temperature is also provided.

## 2 THE PHYSICS BEHIND INFRARED RADIATION

### 2.1 The electromagnetic spectrum

Solar radiation reaching the Earth, also known as the *electromagnetic spectrum*, exhibits a dual nature as it acts both like a wave and a particle traveling in packets of energy (*photons*) which propagate through space at the speed of light (2.998 × 10^8^ m/s).^14^, ^15^, ^16^ The wavelike properties of electromagnetic radiation are described by the relationship of velocity (*c*) to wavelength (*λ*) (the distance between two consecutive peaks of a wave) and frequency (*v*) (number of cycles per second, or hertz, Hz), expressed in the formula^14^:

c=λv

**FIGURE 1:**
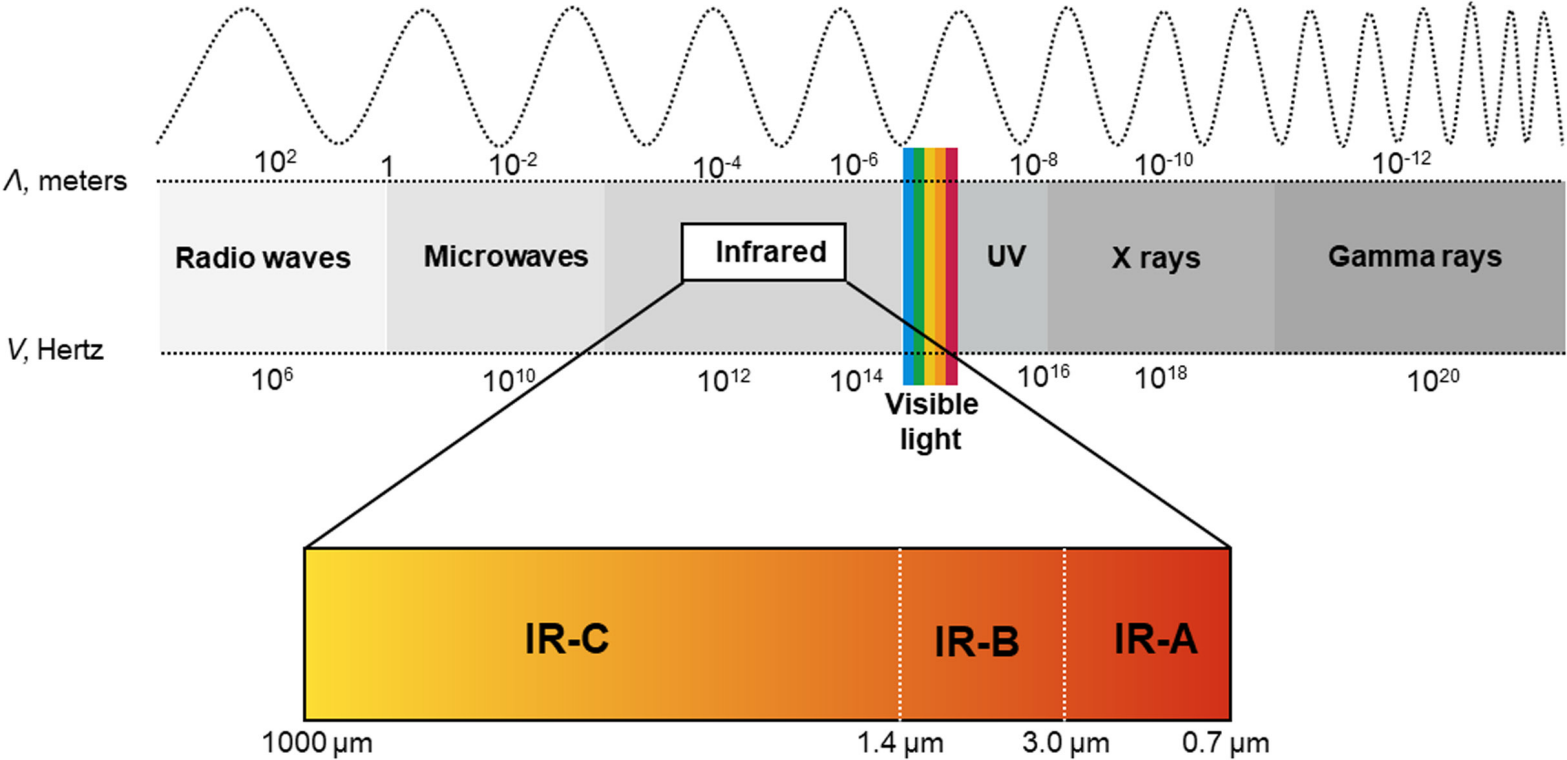
Electromagnetic spectrum and infrared radiation

Regarding photons, the energy carried is described by Planck's equation:

E=hv

where *E* is the energy (given in Joules [J]); *v* is the frequency (Hz); and *h* is Planck's constant (6.626 × 10^−34^ J·s).^14^ The spectrum of electromagnetic radiation ranges from 290 nm to more than 1,000,000 nm,^17^ and is generally divided into seven regions of decreasing wavelength (or increasing energy and frequency). The common designations, as shown in Figure 1, are radio waves, microwaves, infrared (IR), visible light, ultraviolet, X‐rays, and gamma rays.^15^

Infrared radiation constitutes the waveband longer than 0.750 μm and up to 1000 μm.^11^ Corresponding frequencies and quantum energies are 0.3–400 THz and 0.0012–1.65 eV, respectively.^18^ Historically, infrared radiation has been divided into three bands, the definition of which differs across industries. The International Commission on Illumination (CIE) indicates the following nomenclature and ranges: IR‐A (0.7–1.4 μm); IR‐B (1.4–3 μm); IR‐C (3–1000 μm) (Figure 1).^19^ Alternatively, the International Standard Organization (ISO) 20473 provides the following definitions: near‐IR as 0.78 to <3 μm; mid‐IR as ≥3 to <50 μm; far‐IR or FIR as ≥50 to <1000 μm.^20^ In this review, we will be using the CIE definition.

### 2.2 Electromagnetic radiation and nanoscale energy transfer

All matter (solid, liquid, gas) can absorb as well as emit energy in the form of electromagnetic radiation.^21^ The absorption of energy in the visible region of the spectrum excites electrons in molecular bonding orbitals to a higher quantum energy state; this absorbed energy is converted into heat (vibrational energy) and lost either as emitted infrared radiation (radiative heat) or as emitted visible light of a longer wavelength (fluorescence). The energy carried by wavelengths in the infrared region is directly absorbed by molecular vibrational levels and emitted as infrared radiation. For an object with a temperature T (Kelvin) and a surface area (A), the radiative energy transfer in a time *t* is given by the Stefan–Boltzmann law of radiation, where *P* is net radiated power, *e* is emissivity, *A* is radiating area, *T* is temperature of radiator, σ is Boltzmann's constant (σ = 5.6703 × 10^−8^), and *T*_c_ are the temperature of the surrounding matter^22^:

$$P=\mathrm{e\sigma A}\left(T^{4}-T_{C}^{4}\right)$$

**FIGURE 2:**
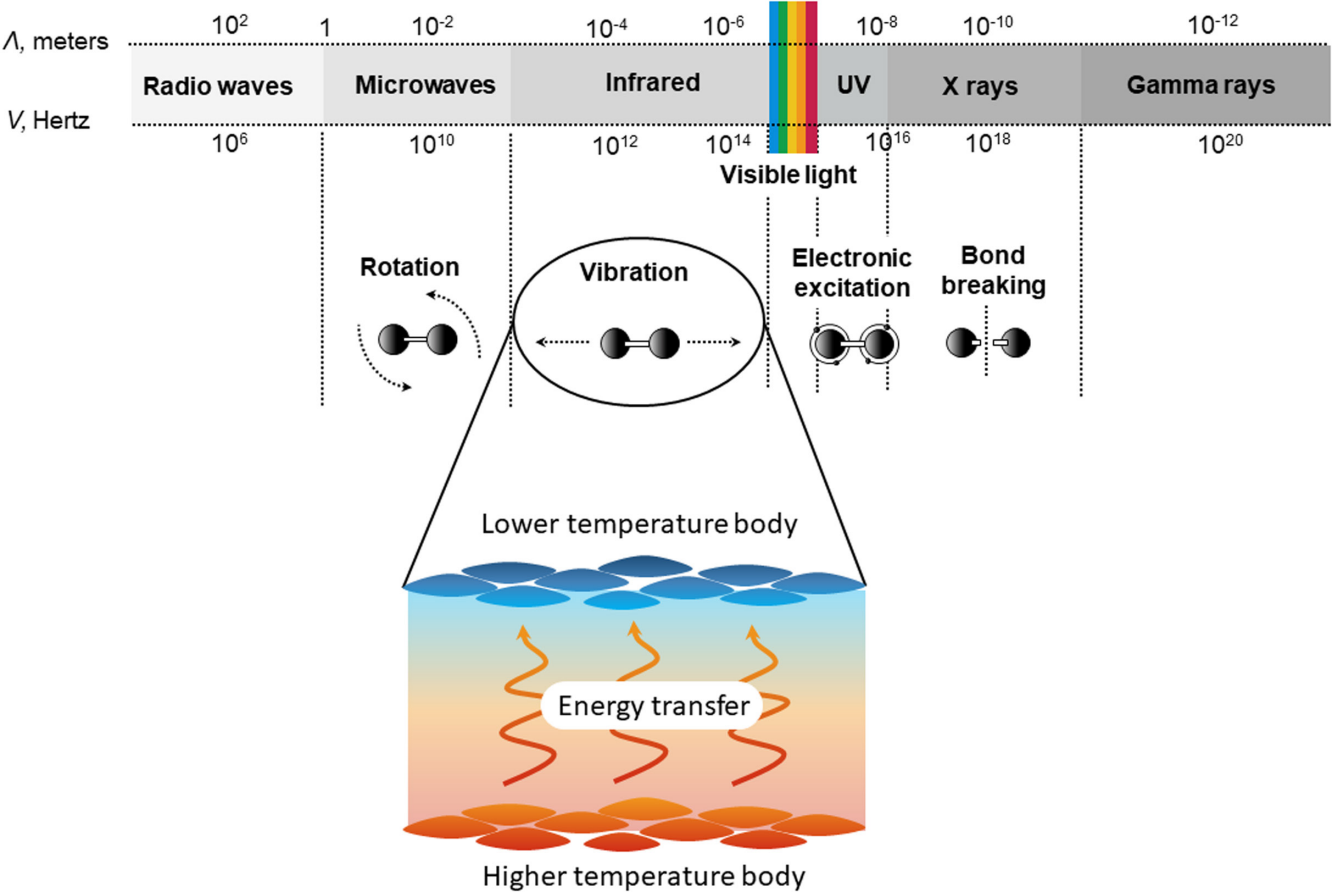
Electromagnetic radiation and energy transfer. Infrared radiation, a nonionizing radiation, transfers enough energy to the receiver to increase the vibrational energy levels of the bonds or molecules, which ultimately result in an increase of temperature. Abbreviations: *λ*, wavelength; *v*, frequency; UV, ultraviolet

Although electromagnetic radiation occurs at all temperatures above absolute zero, the amount of energy (heat) an object can radiate depends greatly on the difference in temperature between the systems involved. In fact, energy transfer occurs from high to lower temperature bodies. It is also important to note that due to the first law of thermodynamics, the internal energy of all systems involved in the radiation (emitter or receiver) changes as a consequence of the energy transfer (energy can neither be created nor destroyed).^23^ Within molecules, internal energy can be stored in two main ways, either by exciting the electronic quantum energy levels to a higher state or by increasing the vibrational, rotational, and translational energy levels of the bonds or molecules. Depending on the amount of energy transferred, radiation can be divided into ionizing and nonionizing. Nonionizing radiation (ultraviolet or visible light) transfers enough energy to the receiver to excite the electron in the highest occupied molecular orbital to the lowest unoccupied molecular orbital. By contrast, the energy carried by ionizing electromagnetic radiation is strong enough to entirely remove tightly bound electrons from an atom or molecule (Figure 2).^16^ Ionizing radiation causes damage to biological matter and living cells; for instance, radiotherapy with high‐energy radiation such as X‐rays or gamma rays is used to destroy tumor cells. Infrared is a type of nonionizing radiation whose absorption leads to changes in the vibrational and rotational energy levels of molecules and bonds.^11^, ^24^ All types of infrared radiation (IR‐A, IR‐B, IR‐C) increase the temperature of the absorbing matter, which extent depends on the power density of the radiation, the absorption coefficient of the material, and the rate of energy lost by emission, convection, or conduction.

Radiative energy transfer at the nanoscale is a relatively new research field that has attracted much attention in the last two decades due to its potential applications.^25^ Infrared nanoscale technology, besides being experimentally more accessible nowadays,^25^, ^26^, ^27^, ^28^ exhibits unusual properties. First, the energy exchange is not limited by the Stefan–Boltzmann law; moreover, electromagnetic radiation at nanoscale is almost monochromatic and it can be spatially coherent.^29^ Energy transfer between surfaces at close vicinity has important applications in nanoscale energy conversion devices, such as emitting ceramics, and studies of its effects may explain some observed positive effects of infrared radiation.

## 3 BIOLOGICAL EFFECTS OF INFRARED

### 3.1 Photobiomodulation

As defined by Anders et al., photobiomodulation therapy is “a form of light therapy that utilizes non‐ionizing forms of light sources […] in the visible and infrared spectrum. It is a non‐thermal process involving endogenous chromophores eliciting photophysical and photochemical events at various biological scales. This process results in beneficial therapeutic outcomes including but not limited to the alleviation of pain or inflammation, immunomodulation, and promotion of wound healing and tissue regeneration.”^30^ Photon absorption converts the radiation energy into biological signals.^31^ All photobiological responses are determined by the absorption of energy by photoacceptor molecules (chromophores) during irradiation. Photobiomodulation effects are thought to be due to two main types of photoacceptors: cytochrome c oxidase and intracellular water.^32^ In the case of infrared, interaction with water in membranes, mitochondria, and/or cells could stimulate signaling pathways, including stress signaling, metabolic processes, cell proliferation/differentiation, and homeostasis.^33^ One additional possible mechanism is the effect of infrared radiation on heat‐sensitive transient receptor potential (TRP) ion channels.^34^ These ion channels (e.g., calcium channels) may be activated by the absorption of infrared radiation by nanostructured water layers (thin layers of water that build up on hydrophobic surfaces such as cellular membranes),^35^ altering the protein conformation at the nanoscale without causing bulk heating of the tissue up to 40°C (which is the required level for TRP activation measured in the laboratory).^34^, ^36^

### 3.2 Penetration of infrared radiation into biological tissue

**FIGURE 3:**
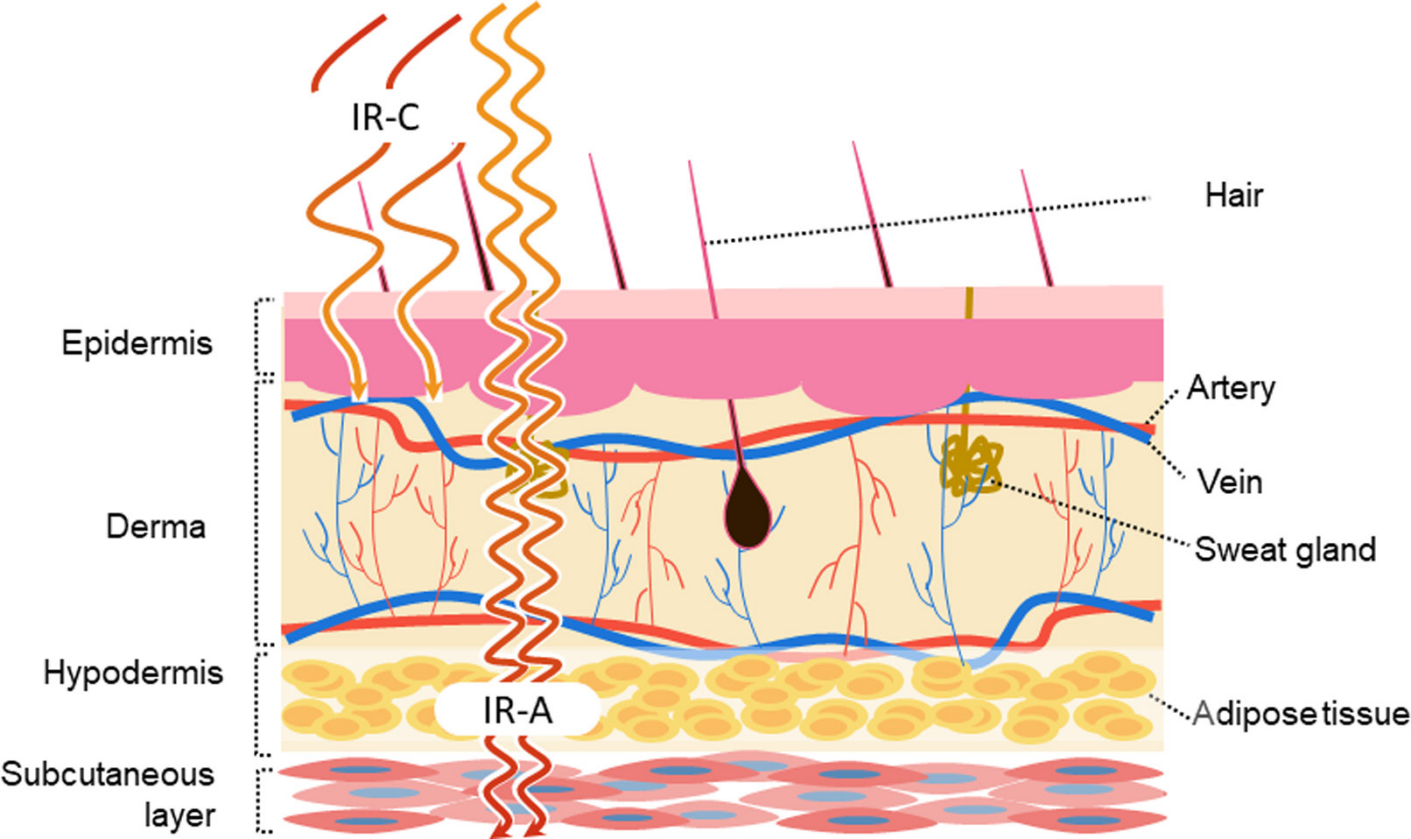
Skin anatomy and infrared penetration depth

Overall, the depth of penetration into the skin and subcutaneous tissue decreases with increasing wavelength in the infrared spectral region.^24^ The deepest skin penetration is obtained with wavelengths between 800 and 850 nm.^37^ Short wavelengths in the IR‐A range reach the subcutaneous tissue (up to 5 mm), whereas IR‐C is absorbed completely in the epidermal layers.^24^, ^38^ Skin anatomy and infrared penetration depth are shown in Figure 3.^39^ It should be noted that infrared penetration has been assessed either via ex vivo tissue studies or using simulation models,^40^, ^41^ thus it is reasonable to assume that results might differ in living human tissue. The experience with the use of infrared heat lamps and infrared saunas using IR‐C radiation suggests that while the penetration might be superficial, the effect initiated by the IR‐C radiation could reach deeper skin layer.^11^ Indeed, externally applied IR‐C light has demonstrated positive therapeutic effects in tissues at considerable depth that very little residual radiant energy could reach (e.g., brain, cardiovascular system, musculoskeletal system).^33^, ^42^, ^43^, ^44^, ^45^ The overall positive effect of the photobiomodulation initiated by IR‐C radiation indicates that there might be a “companion” energy transfer pathway involved.

### 3.3 The hypothesis of water as a dynamic biomolecule

The dominant and most ubiquitous chromophore in nature is water, acting both as an emitter and receiver.^46^ The conventional view of the role of water in biological systems is that of an inert medium in which macromolecules move.^47^ It is becoming increasingly evident that this view is limited in perspective and that liquid water possesses unique properties that make it particularly well suited to participate in the process of energy transfer.^11^ Specifically, water is a well‐known carrier of vibrational and electronic excitation energy, and it is also a particularly strong chromophore in the IR‐C spectral range.^48^, ^49^ Water exhibits high morphological flexibility and is known to form dynamic H‐bonded supramolecular structures and networks within the interfaces and nanospaces of biological tissues.^50^, ^51^ The vibration and excitation properties of these networks may have significant implications in the transfer mechanisms of the electrons and protons underlying the energy dynamics of biochemical reactions. In living systems, also fundamental is the presence of charged groups on proteins, which have the potential to alter further the overall water molecular assembly.^11^ To date, research on photobiomodulation has mainly focused on the importance of light interactions with mitochondrial transmembrane proteins, however, some researchers have begun to consider the potential significance of the water oscillator mechanism.^11^ Human bodies contain up to 70% water and the majority of this water exists in proximity to the biological interfaces of cell membranes and within confined spaces in and around macromolecules, microtubules, channels, and other structures.^52^ It has been found that water cluster size and temperature affect the infrared absorption spectrum significantly.^53^ Experimental studies have shown that energy dynamics of water at interfaces and in confined nanospaces are different to that of free “bulk” water.^51^ Here, water dynamically responds to its environment structurally and energetically, mediating and driving a variety of critical biological functions. There is a growing body of theoretical work and experimental evidence for the active participation of water in cellular and biomolecular dynamics including cell membrane potentials, protein folding, and DNA conformation processes.^54^, ^55^ Many researchers are beginning to consider the possibility that the water oscillator mechanism may contribute or perhaps precede the cytochrome c oxidase pathway for infrared light‐induced biomodulatory activation.^56^, ^57^ However, this hypothesis remains theoretical as, unfortunately, the technology available nowadays does not allow in vivo experiments to be conducted on the effects of IR‐C on tissue water molecules.

## 4 PROPOSED MECHANISMS OF ACTION OF IR‐C

**FIGURE 4:**
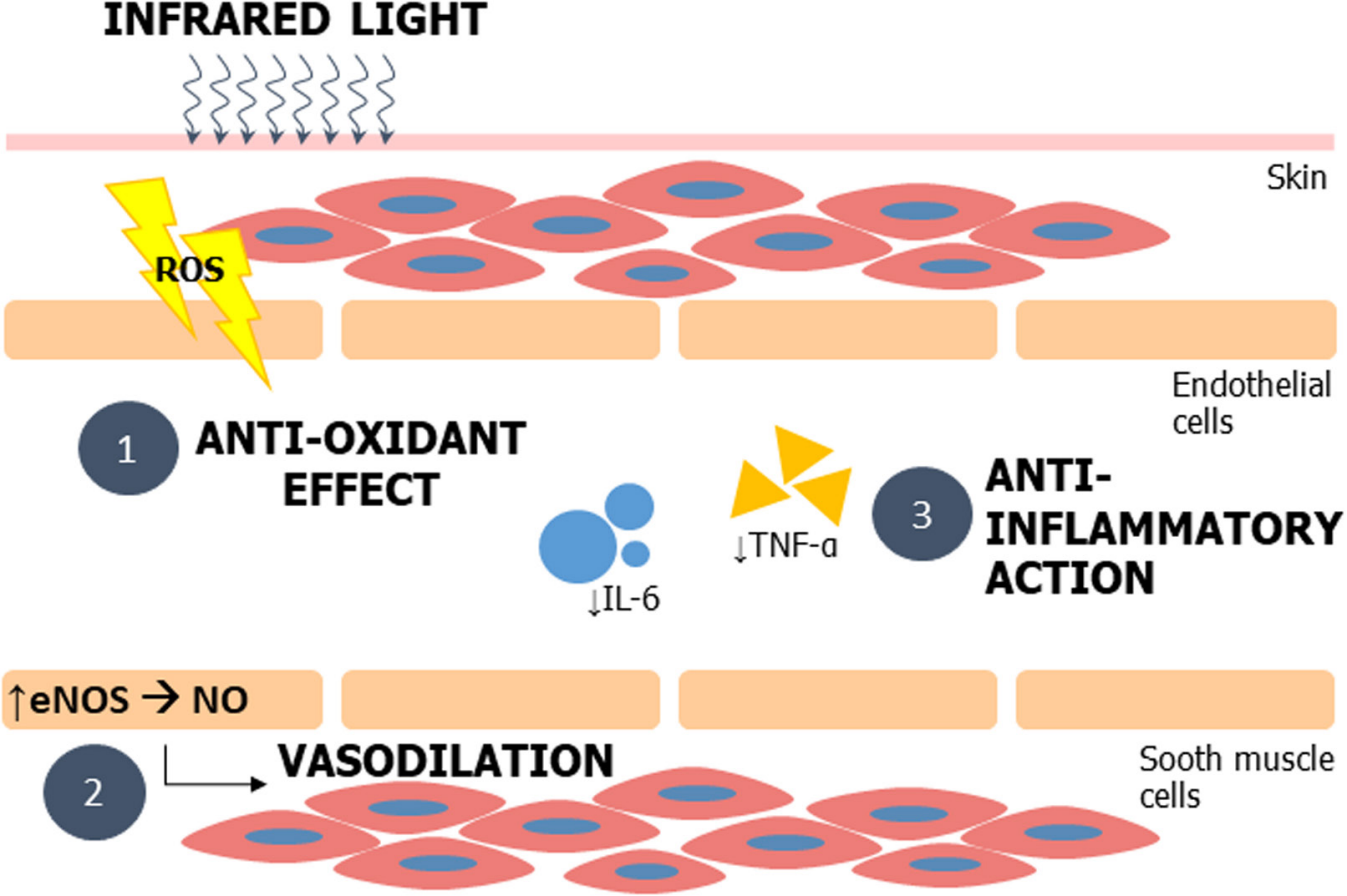
Cellular signaling pathways triggered by IR radiation^58^

In the last decade, experimental and clinical studies have been published describing a variety of physiological effects of IR‐C radiation.^11^, ^13^, ^17^, ^24^ Based on the current evidence, the mechanisms of IR‐C that could explain the improvement in musculoskeletal conditions can be grouped into three categories: (1) upregulation of endothelial nitric oxide synthase (eNOS) and increase in nitric oxide (NO) bioavailability, (2) reduction in oxidative stress, and (3) anti‐inflammatory.^58^ A summary of the possible cellular pathways triggered by infrared radiation is presented in Figure 4 **.**

### 4.1 Upregulation of endothelial nitric oxide synthase and increase in nitric oxide bioavailability

Infrared may directly induce eNOS expression. NO is a crucial vasodilator substance, produced by the eNOS, which acts on smooth muscle cells to induce muscular relaxation.^58^ NO is a paracrine factor that controls vascular tone, inhibits platelet function, prevents leukocyte adhesion, and reduces intimal proliferation.^7^ Many studies have revealed that the biological activities of irradiation with IR‐C are associated with the eNOS/NO pathway.^10^, ^59^ In the short term, the thermal effect of IR‐C can cause vasodilation, which reduces laminar shear stress (the stress that occurs in vascular tissue due to blood flowing past vessel walls). In response to this temporary vasodilation, the cardiac output increases and consequently the peripheral blood flow, leading to an actual increase in peripheral vascular resistance, which is one of the most important stimuli for eNOS expression, and therefore further production of NO. However, the increased eNOS expression through increased shear stress appears to be only partly due to a temperature effect, as IR‐C studies have shown that the beneficial effects of IR‐C on endothelial function persist beyond this temporary rise in local temperature.^8^, ^9^

In vitro and animal studies have reported the increase in expression of eNOS following IR‐C irradiation and suggest that upregulating NO production by increasing eNOS expression level, either directly or indirectly, is a critical mechanism by which IR‐C therapy improves endothelial function. Akasaki et al. found that repeated IR‐C sauna therapy over 5 weeks could induce angiogenesis by upregulating eNOS expression in mice with hind limb ischemia.^60^ Miyauchi et al. reported very similar results after 4 weeks of IR‐C sauna therapy, a significant increase of eNOS expression and NO production in mice with hind limb ischemia.^10^ In addition, they noticed an increase of the heat shock protein 90 (HSP90), a chaperone protein produced in response to stress conditions, which forms a complex with Akt/PKB resulting in phosphorylation and activation of eNOS.^58^ The addition of a HSP90 inhibitor reduced the effect induced by IR‐C, suggesting a potential additional mechanism of action.^10^ Hsu et al. evaluated the biological effect of IR‐C on vascular endothelial growth factor‐induced proliferation in human umbilical vein endothelial cells (HUVECs) and found that a nonthermal effect of IR‐C induced translocation of the transcription factor promyelocytic leukemia zinc finger (PLZF) to nuclei, and ultimately inhibited vascular endothelial growth factor‐induced proliferation in HUVECs via the phosphoinositide 3‐kinase/Akt signaling pathway.^7^ In addition to enhancing eNOS expression, IR‐C may increase NO production via phosphorylation of eNOS by promoting the Ca^2+^/calmodulin‐dependent protein kinase II‐NOS.^61^ Moreover, it has been shown that the heat‐sensitive TRPV3 ion channel mediated the production of NO in the skin via nitrite reduction, and this was independent of NOS enzyme activity.^62^

NO has a number of physiological effects that go beyond the regulation of the blood flow to tissues, and that may be involved in the pathogenic mechanisms of musculoskeletal pain. NO is also a mediator of muscle satellite cell activation, fundamental in the initiation of muscle regeneration after injury.^63^, ^64^ For instance, in vitro studies showed that pharmacological inhibition of NOS activity reduced the extent of muscle repair, thus increasing scarring.^65^ Moreover, muscle‐derived NO can inhibit the migration of inflammatory cells, thus protecting the muscle from damage caused by an inflammatory response.^66^ NO has been shown to contribute to both inflammatory and neuropathic pain in the periphery.^67^ A possible mode of action of NO as a peripheral antinociceptive agent is the opening of ATP‐sensitive K^+^ channels in the membrane of nociceptors which leads to hyperpolarization and thereby reduces excitability.^67^

### 4.2 Antioxidant effects

Oxidative stress, defined as an excess production of reactive oxygen species (ROS) maintained for some time, plays a critical role in pathological conditions resulting in oxidative damage.^58^ The antioxidant effects of infrared therapy have been investigated specifically for the treatment of cardiovascular conditions, and may explain the benefits of Waon therapy. Fujita et al. conducted a study in 40 patients suffering from chronic heart failure (CHF) and found that after 4 weeks of Waon therapy, concentrations of plasma hydroperoxide (an index of oxidative stress) decreased significantly (*p* < .001) and the nitric oxide metabolites increased (*p* < .05).^9^ In contrast, none of these variables changed over the 4‐week interval in the control group. Furthermore, animal experiments were performed using TO‐2 hamsters (who are prone to develop cardiomyopathy). Immunohistochemistry analysis showed cardiac expression of 4‐hydroxy‐2‐nonenal, a marker of oxidative stress, was also decreased during treatment compared to the control group. Western blotting analysis showed that cardiac expression levels of HSP27, manganese superoxide dismutase, and HSP32, which reduce oxidative stress, were significantly upregulated.^9^ Masuda et al. reported that repeated infrared sauna therapy could protect from oxidative stress (lipid peroxidation) in patients at risk of atherosclerosis.^68^ The study group investigated the effect of 15 min of infrared sauna, once a day for 2 weeks, on the urinary levels of 8‐epi‐prostaglandin F2α (PGF2α), a reliable marker of oxidative stress in vivo. Epi‐PGF2α levels in the sauna group were significantly lower than those in the nonsauna group at 2 weeks after admission.^68^ Finally, oxidative stress and inflammatory biomarkers were measured in plasma of patients with intermittent claudication treated with IR‐C‐emitting bioceramic wraps. The intervention induced a decrease in oxidative stress, with significantly lower levels of thiobarbituric acid reactive substances (TBARS), as well as an increase in superoxide dismutase and catalase enzyme activities; whereas no significant difference was reported in the evaluation of inflammatory markers.^69^ Because ROS can inactivate NO, thus reducing the bioavailability of NO, a reduction in oxidative stress may also indicate an improvement in endothelial function through an increase in NO production.^58^

Oxidative stress may alter nociception and cause hyperalgesia with local mechanisms.^70^ The reactive oxygen species that occur as a result of oxidative stress cause tissue damage and inflammation, which then increase the stimulation of sensory neurons that play a role in the transmission of pain.^71^ By reducing the amount of oxidative stress, infrared may help to relieve pain and inflammation at the muscular level.

### 4.3 Anti‐inflammatory effects

Infrared therapy may aid in reducing the level of inflammation. In preclinical studies, Chang et al. investigated the anti‐inflammatory effect of IR‐C treatment in mice with lipopolysaccharide (LPS)‐induced peritonitis by measuring the plasma levels of interleukin‐6 (IL‐6), tumor necrosis factor alpha (TNF‐α), and endothelial nitric oxide synthase (eNOS). Cytokines IL‐6 and TNF‐α play key roles in the inflammatory cascade and have been recognized as targets of therapeutic intervention. Additionally, the RNA level of peripheral blood mononuclear cells was analyzed to capture the expression of IL‐6, TNF‐α, and eNOS. The group found that IR‐C treatment significantly inhibited plasma IL‐6 levels (after 30 min) and TNF‐α RNA concentrations (at 2 h) compared to control. Moreover, the initial decrease in eNOS synthase‐RNA recovered after 2 h, whereas in the control group eNOS synthase‐RNA continuously decreased.^72^ A similar study was conducted on a rabbit model of LPS‐induced knee arthritis. Positron emission tomography scanning of fluorodeoxyglucose isotopes showed that IR‐C therapy was capable of relieving inflammation in the joints at 7 days after LPS injection.^73^ The same group also studied the effect of IR‐C on prostaglandin E2 (PGE2) in vitro in cells treated with LPS. The anti‐inflammatory effect was further confirmed by the PGE2 inhibition.^73^ Lin and colleagues studied the effect of IR‐C on vascular inflammation in HUVECs. They found that IR‐C therapy exerted a potent anti‐inflammatory effect via the induction of heme oxygenase‐1, an enzyme that plays a critical role in the prevention of vascular inflammation. In clinical studies, IR‐C radiation also inhibited the expression of several circulating inflammatory markers, including IL‐8.^74^ Finally, lower serum levels of IL‐6 and endothelin‐1 (ET‐1) following IR‐C therapy were reported in patients during the postoperative period after total knee arthroplasty.^75^

Inflammation can increase the pain response to noxious stimuli through the sensitization of sensory afferent fibers. During inflammation or tissue injury, damaged cells and immune cells release inflammatory mediators, such as bradykinin, PGE2, proinflammatory cytokines (IL‐1β, IL‐6, TNF‐α). TNF‐α could be a direct cause of pain, since it may promote the sensitization of nociceptors which leads to chronic pain and muscle fatigue.^76^, ^77^ Elevated serum levels of IL‐8 and IL‐6 have also been demonstrated to induce hyperalgesia, fatigue, and pain.^78^, ^79^, ^80^ These inflammatory mediators act both directly on peripheral nociceptors, eliciting sensitization, and indirectly by promoting inflammation and the release of prostaglandins. Following acute inflammation, a significant percentage of nonmyelinated nerve fibers, otherwise insensitive to stimulation in the normal joint, develop responsiveness to mechanical stimulation and exhibit increased activity.^4^ Thus, by increasing NO levels and reducing oxidative stress and inflammatory mediators, infrared can indirectly relieve pain.

## 5 BIOCERAMICS

There are several ways to deliver infrared radiation for therapeutic use, varying from heat lamps or saunas – that require an external power source – to infrared‐emitting materials that rely solely on body heat as a source of power.^11^, ^13^ Bioceramic describes a specific type of mineral material that emits IR‐C radiation at body temperature, and which can produce biological effects on the tissue, particularly when worn in close contact for extended periods of time.^81^, ^82^ While the power density emitted by these fabrics is very small when compared to electrically powered IR sources, this is compensated by the fact that garments and patches can be worn for extended periods of time (hours or days), while lamps or saunas are usually only used for minutes at a time. Bioceramic materials are produced by a combination of polymers with ceramic‐containing mineral oxides like silicon dioxide (SiO_2_), aluminium oxide (Al_2_O_3_), and titanium dioxide (TiO_2_).^11^ In industrial applications, these minerals are often used in the construction of firebricks and gas mantles. In domestic kitchens, the use of a clay cooking pot is often preferred to a metal cooking pot because of its ability to emit more infrared radiation at lower temperatures. There have been some attempts to characterize the properties of these infrared‐emitting fabrics in the laboratory, including reflectance, transmittance, and emissivity. Emissivity is a measure of how much radiation (7.5–14 μm) an object can absorb and emit compared with a black body (a body that absorbs and emits all radiation falling in it), whose emissivity is defined as 1.0.^83^ Emissivity is a surface phenomenon, therefore nanoparticles or microparticles (which have a large ratio of surface area to mass) are considered to be the most efficient configuration for emitting infrared radiation compared to bulk ceramic material. Anderson et al. utilized Fourier transform infrared (FTIR) spectroscopy to measure the spectral optical properties of textile fabrics woven with varying percentages of ceramic particle‐bearing polymeric fibers, and found that the emissivity of polyester fabric can be engineered controllably via the inclusion of ceramic microparticles within the fabric fibers.^84^, ^85^

In general, the mechanism of action of infrared radiating materials is to absorb heat energy from the body (radiation, convection, and conduction) and maintain the temperature at sufficiently high levels to be able to reemit the IR‐C energy back to the body with a broad peak centered at 10 μm, according to the Stefan–Boltzmann law.^11^, ^86^

## 6 INFLUENCE OF BIOCERAMICS ON BLOOD CIRCULATION

The effects of infrared‐emitting ceramics on skin perfusion and blood flow have been investigated by analyzing the changes observed on several biomarkers. For instance, in a study conducted on 153 healthy individuals wearing shirts containing ceramics compared with standard polyester shirts, changes on skin temperature, arterial oxygen saturation, and transcutaneous partial pressure of oxygen (tcPO_2_) were assessed. Of note, the emitting garment was associated with a significant increase in tcPO_2_ and arterial oxygen saturation levels, but not an increase of skin temperature, supporting the hypothesis that vasodilatory effects of infrared are independent of temperature variations.^87^ Similar findings were observed in another study testing the relation between increased blood flow (measured as tcPO_2_ changes) and improved muscular performance (measured as mean hand grip strength). Individuals wearing a shirt containing quartz, silicon oxide, and titanium oxide particles showed a tcPO_2_ increase, but not an increase in skin temperature. Nevertheless, improved skin oxygenation was associated with higher grip strength, suggesting that infrared‐emitting garments could be used in athletic settings.^86^ The benefits of infrared on blood circulation were supported further by a study on patients with Raynaud's syndrome, a condition characterized by attacks of limited blood supply to specific sites of the body (e.g., fingers and toes). Ninety‐three patients were given ceramic‐embedded gloves and after a 3‐month follow‐up, significant improvements were observed in the active group compared with control. Although direct measures of blood flow and oxygen levels were not captured, reduced pain and disability of the arm, shoulder, and hand were recorded among those wearing infrared‐emitting gloves, providing encouraging results for the use of such garments.^88^ The topical use of infrared‐emitting ceramic also showed blood circulation benefits in the treatment of edema of the limb inferior extremities. Over a period of 28 days, both the extent of edema and pain were reduced in the group wearing compressive bioceramic socks compared with control.^89^

## 7 MUSCULOSKELETAL APPLICATIONS OF BIOCERAMICS

Several studies have evaluated the property of IR‐C‐emitting ceramics to reduce different types of pain. Infrared‐emitting nanoparticles have recently been incorporated into apparel, such as gloves, socks, belts, or patches, to provide an easy and practical application for their use.^82^, ^90^ A summary of studies that have investigated the effect of IR‐C radiation on pain, muscular activity, or musculoskeletal conditions is shown in Table 1.

**TABLE 1:** Studies investigating the effect of IR‐C‐emitting ceramics on muscular activity and pain perception

Medical application	Author	Population	Light source or material (garment type)	Key results
Exercise performance and recovery	Leung et al.^102^	Skeletal muscles (frog)	Bioceramic (bracelet)	Promoted stimulation of the muscle for a long period of time
	Leung et al.^73^	Nonathletes	Bioceramic (bracelet)	Activated parasympathetic responses in resting or after exercise, reduce resting metabolic rate
	Loturco et al.^90^	Soccer players	Bioceramic (socks)	Reduced delayed‐onset muscle soreness at 48 hours and 72 hours after moderate and intense exercise
	Mantegazza et al.^58^	Healthy subjects	Bioceramic (sports clothes)	Increased exercise capacity and delayed anaerobic metabolism
	Nunes et al.^82^	Futsal players	Bioceramic (sleep clothes)	Facilitated recovery during the early phases of an intensive training period
Arthritis	Bagnato et al.^91^	Patients with knee osteoarthritis	Bioceramic adhesive plasters	Decreased pain scale scores between weeks 1 and 4
	Djuretić et al.^103^	Rats with collagen‐induced arthritis	Bioceramic cage bedding	Reduced inflammatory arthritis
	Leung et al.^72^	Induced arthritis (rabbit)	Bioceramic	Reduced inflammatory arthritis and maintained bone health
	York and Gordon^92^	Patients with diabetes and neuropathy	Bioceramic (socks)	Reduced pain, though not significantly
Reduction of pain	Santos et al.^104^	Patients with fibromyalgia	Bioceramic (shirt)	Reduced pain
	Da Silva et al.^69^	Intermittent claudication	Bioceramic (wraps)	Improved oxidative stress profile
	Lai et al.^105^	Patients with chronic myofascial neck pain	Bioceramic (neck device)	Reduced muscle stiffness
	Lee et al.^93^	Patients with primary dysmenorrhea	Bioceramic (belt)	Reduced pain
	Salm et al.^106^	Patients with fibromyalgia	Bioceramic (shirt)	Reduced pain

Bagnato et al. evaluated the efficacy of an IR‐C‐emitting plaster in the treatment of knee osteoarthritis (OA) in a randomized, placebo‐controlled clinical study.^91^ The plaster was applied for 12 h a day for 5 days per week for a total of 4 weeks of treatment. The primary endpoint was to assess pain improvement from baseline to week 4 post treatment using a visual analog score (VAS). Pain score decreased significantly during treatment in the IR‐C group (*p* < .01), while no significant difference was found in the placebo group. Additionally, ultrasound showed that patients from the IR‐C group had less joint effusion (40%) compared to baseline (80%), while no changes were seen among the placebo group.^91^ Loturco et al. investigated the effects of IR‐C‐emitting noncompressive pants on indirect markers of exercise‐induced muscle damage and physical performance recovery in soccer players. They found that the use of IR‐C‐emitting clothes during a 10‐h sleeping period over three successive nights contributed to reduced delayed‐onset muscle soreness at 48 h and 72 h after exercise.^90^ The use of IR‐C‐emitting socks also appeared to have a beneficial impact on chronic foot pain resulting from diabetic neuropathy or other disorders. Participants of a double‐blind randomized trial completed several questionnaires for analyzing pain reduction over 4 weeks (VAS, Brief Pain Inventory [BPI], McGill Pain Questionnaire [MPQ], SF‐36). More pain reduction was reported by those treated with emitting socks than controls.^92^ The efficacy of an IR‐C‐emitting sericite (a common mineral) belt in patients with primary dysmenorrhea was evaluated over three menstrual cycles (and two follow‐up cycles) by Lee et al. in a multicenter, randomized, double‐blind, placebo‐controlled trial (*n* = 104). The main outcome measures were the severity of dysmenorrhea assessed by a 10‐point visual VAS and the number of patients who took analgesics at each menstrual cycle. Although the severity of dysmenorrhea gradually decreased during the treatment period in both groups, the follow‐up period showed a constant decrease of VAS scores in the experimental group, whereas the VAS score gradually returned to baseline in the control group. These data suggested the potential long‐term benefits of IR‐C‐emitting treatment.^93^ The ability of IR‐C irradiation to alleviate pain was further reported in a study by Lai et al. which concluded that short‐term treatment using an IR‐C‐emitting neck device partly reduced muscle stiffness in chronic neck pain. Although differences in VAS and pressure–pain threshold scores for the experimental and control groups were not statistically significant, the improvement in muscle stiffness in the experimental group warrants further investigation of the long‐term effects of IR‐C treatment for pain management.^94^ Of note, few of the randomized studies presented also investigated the safety and tolerability of the infrared‐emitting sources used during the studies. The study groups lead by Bagnato, Lee, and Lai, all reported no serious adverse events related to infrared radiation exposure.^91^, ^93^, ^94^ Overall, different infrared‐emitting materials (ceramics and fabrics) have been recognized as well tolerated, and skin irritation and itching (which disappeared within a few days and without treatment) were the most common effects associated with infrared exposure via emitting garments.^88^, ^93^ These safety results are not surprising, since the power density achieved by IR‐C‐emitting ceramics and fabrics is too low to pose any safety concern.^17^ However, it should be noted that irradiation has a dose‐dependent effect, and high levels of IR‐A can pose risks to the skin.^17^

## 8 DISCUSSION AND CONCLUSIONS

Musculoskeletal pain is a common condition among the general population and the leading cause of disability worldwide.^1^ Inadequately managed, musculoskeletal pain can reduce mobility, impair quality of life, and increase healthcare costs. Multidisciplinary approaches are fundamental for the effective management of musculoskeletal conditions.^95^ Although pharmacological therapy is important to enhance patient recovery, drug therapy still has limitations. For instance, NSAIDs may cause significant stomach ulcers and opioids have long been recognized for their addictive side effects.^95^ According to a statement of the Drug Enforcement Administration (2018), 6.2 million people abuse prescription drugs.^96^ Also of note is that conventional therapies often treat the symptoms of the disease without effectively addressing the underlying causes.^97^ A reduced reliance on conventional analgesic medicines can help limit the adverse effects. Therefore, new approaches to pain management have emerged over the past years. Recent guidelines recommend the implementation of preventative strategies and physical tools to minimize the use of medications.^95^ Noteworthy, people suffering from musculoskeletal pain usually opt for self‐management options of their symptoms, thus a multidisciplinary approach to treatment may improve the outcome.^98^ In this therapeutic context, infrared therapy can offer the benefits of reducing the discomfort caused by pain and inflammation, while promoting the body's natural healing mechanisms.^13^, ^99^, ^100^, ^101^ Overall, infrared therapy has been recognized as an effective, well–tolerated, and nonpharmacological option for improving health.^11^ The absorption of infrared radiation has been shown to stimulate signaling pathways related to pain perception. The presence of water molecules may play a key role in determining the penetration depth and the biological effects of the infrared radiation.^11^ In this article, the increase of nitric oxide levels, reduction of oxidative stress, and inflammatory mediators induced by infrared have been proposed as potential mechanisms explaining musculoskeletal pain relief. Few of the randomized clinical trials investigating the effect of IR‐C‐emitting ceramics on muscular activity and pain perception have also reported their safety. In conclusion, the use of infrared radiation technology is growing, however, the exact molecular mechanism remains elusive and further research is needed to assess and validate current theories.

## AUTHOR CONTRIBUTIONS

JK and JM have made substantial contribution to the acquisition of the literature and analysis. MRH made substantial contribution to the concept of the manuscript and interpretation of the literature. All authors have contributed to drafting, reviewing, and approving the manuscript.

## CONFLICT OF INTEREST

JK and JM have no conflict of interest to declare. MRH declares the following potential conflicts of interest: Scientific Advisory Boards – Transdermal Cap, Inc., Cleveland, OH; Hologenix, Inc., Santa Monica, CA; Vielight, Toronto, Canada; JOOVV, Inc., Minneapolis‐St. Paul, MN; Consulting – USHIO Corp, Japan; Sanofi‐Aventis Deutschland GmbH, Frankfurt am Main, Germany. IIT, HK, and EK are employees of Sanofi CHC and may hold shares and/or stock options in the company.

## Data Availability

Data sharing is not applicable to this article as no new data were created or analyzed in this study.
